# Urine in Good Hands: A Case Report of MGRS type I Cryoglobulinemia

**DOI:** 10.51894/001c.144631

**Published:** 2025-09-30

**Authors:** Catherine Raymond

**Affiliations:** 1 Department of Internal Medicine Henry Ford Warren

## 116

### INTRODUCTION

Monoclonal gammopathy of renal significance (MGRS) is a hematologic disease, causing kidney disease with clonal proliferation of immunoglobin-producing B-lymphocytes or plasma cells (1-11). MGRS is restricted to only renal involvement, leading to misdiagnosis and under treatment. Importance of early diagnosis is paramount due to severity of renal involvement and complicated management. Despite disease severity, there is limited data on optimal treatment. We present a case of MGRS type I cryoglobulinemia causing acute kidney injury.  

### CASE PRESENTATION

Patient is a 57-year-old male with chronic kidney disease stage IIIb, presented to ED by nephrologist’s request for concerns of elevated creatinine outpatient with nephrotic range proteinuria, microscopic hematuria, and complaints of foamy urine and nocturnal urinary frequency. Inpatient workup confirmed acute kidney injury with nephrotic range proteinuria. A 24-hour urine protein showed 12 grams proteinuria but negative serology and infectious workup. Serum electrophoresis showed IgM monoclonal gammopathy with urine electrophoresis having a biclonal IgG Kappa, without urinary free light chains. Renal biopsy showed membranoproliferative glomerulonephritis with monoclonal IgG1 Kappa deposits, representing type I cryoglobulinemic glomerulonephritis or proliferative glomerulonephritis with monoclonal immune deposition (PGMID). Bone marrow biopsy showed occasional CD138+ plasma cells at 4%, excluding multiple myeloma without clonal B cell or plasma cell population. 

Patient completed Rituximab 375mg/m2 weekly with prednisone 40mg daily and improvement of creatinine and proteinuria.

### DISCUSSION

MGRS is a type of monoclonal gammopathy (MG) that causes renal end-organ damage but is not associated with malignancy (9, 10). Previously, it was difficult to diagnosis due to other MGs types having similar presentations but without end-organ damage, such as monoclonal gammopathy of undetermined significance (MGUS) (1, 4, 9, 10). Treatment is often delayed due to the unclear diagnosis. However, given poor renal outcomes, treatment options have been researched (5, 8). Current data (2, 5, 6) indicates therapy should target underlying plasma cell and/or B cell clones. Therapy options targeting plasma cells include bortezomib, cyclophosphamide, and dexamethasone MC, daratumumab-based therapy, and lenalidomide (2). Therapy targeting B cells include rituximab. Our case is an example, highlighting importance of early diagnosis and treatment with rituximab.

